# Characterization of two proline-rich proteins involved in silicon deposition in *Cucummis sativus*

**DOI:** 10.3389/fpls.2025.1664009

**Published:** 2025-08-28

**Authors:** Hao Sun, Feijuan Gao, Xiaoping Kong, Zhen Jiao, Zhongfang Tan, Jie Wu, Bochao He, Yaoke Duan

**Affiliations:** ^1^ National Key Laboratory of Cotton Bio-breeding and Integrated Utilization, School of Agricultural Sciences, Zhengzhou University, Zhengzhou, China; ^2^ Henan Key Laboratory of Ion-Beam Green Agriculture Bioengineering, School of Agricultural Sciences, Zhengzhou University, Zhengzhou, China; ^3^ Shaanxi Engineering Research Center for Vegetables/College of Horticulture, Northwest A&F University, Yangling, China; ^4^ Xining Vegetable Technical Service Center, Xining, China; ^5^ College of Agriculture and Animal Husbandry, Qinghai University, Xining, China

**Keywords:** silicon deposition, proline-rich protein, cucumber, biogenic silica, silicification

## Abstract

**Introduction:**

Silicon can exert benefits on plants when they are suffering stresses, and the benefits are more obvious in high silicon accumulators. However, the molecular mechanism how silicon deposits in plants is not fully understood.

**Methods:**

This study identified the CsPRP family genes in cucumber, and analyzed their functions in cucumber silicon deposition via expression in Escherichia coli. Additionally, their intracellular localization was analyzed via transient expression of green fluorescent protein (GFP) fusion constructs in onion epidermal cells, and their expression profiles were characterized using ProCsPRP1::GUS and ProCsPRP3::GUS transgenic Arabidopsis.

**Results and Discussion:**

Seven PRP genes were identified in cucumber, of which CsPRP1 and CsPRP3 were identified as tandem duplication, CsPRP4 and CsPRP5 were identified as segmental duplication. The binding experiment of silicon showed that both CsPRP1 and CsPRP3 exhibited significant binding characteristics to silicon, but their optimal pH values were different. Transient expression in onion epidermal cells revealed that CsPRP1 and CsPRP3 were specifically localized on the cell wall. Staining of ProCsPRP1::GUS and ProCsPRP3::GUS transgenic Arabidopsis demonstrated that during the seedling phase, CsPRP1 and CsPRP3 were mainly expressed in the mature leaves and roots, and in the mature phase, they were mainly expressed in the leaves, roots, petals and stamens. These results may aid further research into the biological function of cucumber PRP and the molecular mechanism of silicon deposition in cucumber.

## 1 Introduction

Being one of the most abundant elements on Earth, silicon has been observed to be advantageous to plants growth and productivity, especially when they are suffering adversities like drought, salinity, heavy metal, extreme temperatures, and nutritional imbalances (Marschner, 1995; Debona et al., 2017; Coskun et al., 2019; Leroy et al., 2019; Ahanger et al., 2020; Li et al., 2025). Both symplast and apoplast pathways have been employed by plants in the uptake and transport of silicon. Symplast pathway is driven by passive diffusion and apoplast transmembrane transport is mediated by transporters including Lsi1, Lsi2, Lsi3 and Lsi6, which demonstrated influx or efflux transport activity for silicon and localized at specific tissues with or without polarity distribution (Ma et al., 2006, 2007; Yamaji et al., 2008, 2015; Sun et al., 2017, 2018; Huang et al., 2022; 2025).

Though all plants can uptake silicon passively and/or actively from soil, they accumulated silicon distinctively from 0.1-10% of the shoot dry weight (Epstein, 1999), which is determined by the ability of root to uptake silicon (Mitani and Ma, 2005). Lycopodium and early evolved ferns possess a higher accumulation of silicon, whereas gymnosperms evolved lately have a lower silicon content. Among angiosperms, most monocotyledon accumulated more silicon than the dicotyledon. The Anadactylus, Gramineae and Areca have a higher silicon content, with Gramineae and Cyperaceae having the highest silicon content (Ma and Takahashi, 2002; Trembath-Reichert et al., 2015). The advantage of silicon in enhancing plant resistance is associated with the accumulation of silicon, and the beneficial impact of silicon on plants under stress is more evident in plants with a high silicon accumulation (Mitani-Ueno et al., 2016). Upon absorption by plant roots, silicon is transported via xylem runoff to the plant shoot, where it is stored in the form of hydrated silica (SiO_2_·nH_2_O) in cell walls and intercellular spaces (Epstein, 1994; Zhang et al., 2013). In particular, the silicon deposition in plants such as selaginella and Arundo donax is specialized into phytoliths of various morphologies (Piperno, 2006; Chauhan et al., 2011; Lopes and Feio, 2020; Jia, 2023).

The deposition of silicon in plants employs multiple pathways affected by species, leaf age, plant growth stage and silicon availability in soil (Motomura et al., 2004; Schaller et al., 2022). In cucumber, transpiration rates and the balance between silicon supply and plant demand influence the degree to which active and passive processes are involved in silicon accumulation (Faisal et al., 2012). Kumar et al. (2017) and Kumar and Elbaum (2018) observed that as the silicified wall increased in thickness during silicon deposition, the functional cytoplasm was compressed into a very limited area. Silica deposition occurs in the paramural space of live silica cells, without causing their demise. This suggests that the deposition of silica in leaves is an active, physiologically regulated process, rather than a mere precipitation. Recently, Zexer and Elbaum (2022) observed that the presence of active silicification zones (ASZs) was essential for the accumulation of silica in sorghum roots and silicification is augmented in oxidative stress conditions due to the augmented deposition of lignin-like substances in the ASZs. However, the molecular mechanism of silicon deposition in plants still remains largely to be investigated.

Carbohydrates, callose, proteins, lipids, phenolic compounds, and metal ions have been suggested to be involved in the accumulation of silicon (Law and Exley, 2011; Zhang et al., 2013; Brugiére and Exley, 2017; Guerriero et al., 2018; Kumar et al., 2021; Zexer and Elbaum, 2022). For example, Kumar et al. (2020) identified a protein, Siliplant1, which is rich in proline, lysine, and glutamic acid and is capable of precipitating silica in sorghum (*Sorghum bicolor*) silica cells. Cucumber is a well-known vegetable crop and a typical high-silicon accumulator among dicotyledonous plants (Sun et al., 2017; 2018). Kauss et al. (2003) isolated a proline-rich protein, Csa2G176690, from the hypocotyl of cucumber which was induced by cucumber anthracnose and was observed to induce the precipitation of silicon in silicic acid solution. Nevertheless, no more information about its role in cucumber silicon deposition is available. In this study the *CsPRP* family genes in cucumber were identified, and their functions in cucumber silicon deposition were investigated. This study may help investigate the biological function of cucumber PRP and the molecular mechanism of silicon deposition in cucumber.

## 2 Materials and methods

### 2.1 Identification and characterization of *PRP* genes in cucumber

To identify the *PRP* genes in cucumber, the annotated cucumber genome (cv. 9930, Li et al., 2019) was downloaded from the website of National Centre for Biotechnology Information (http://www.ncbi.nlm.nih.gov, accessed on 22 December 2021).

The sequences of all known PRPs proteins in Arabidopsis (At1g54970 (AtPRP1), At2g21140 (AtPRP2), At3g62680 (AtPRP3) and At4g38770 (AtPRP4)) were used as queries to search the cucumber genome by BLASTp. Upon finding the candidate sequences, they were then tested against the SWISS-PROT database to determine if the identified PRP family was closely related to other plants.

To obtain the sequence length, molecular weight, and isoelectric point information of the identified PRP proteins, the ExPASy online tool (https://web.expasy.org/protparam, accessed on 3 January 2022) was utilized. Additionally, the PLANT-PLOC online tool, accessed on 6 January 2022) was employed to predict the protein subcellular localizations (Chou and Shen, 2007). The signal peptides and their cleavage sites were predicted by SignalP - 6.0 (https://services.healthtech.dtu.dk/services/SignalP-6.0/, accessed on 24 March 2023).

### 2.2 Phylogenetic relationship, gene, protein structure and protein motif analysis

A phylogenetic tree was constructed using MEGA-X and the maximum likelihood method with Poisson model and 1000 bootstrap replications (Kumar et al., 2018). PRP protein sequences of Arabidopsis and rice were obtained from UniProt website (https://sparql.uniprot.org, accessed on 15 January 2022). The gene structures of CsPRPs were analyzed by gene structure display server (GSDS) online program (http://gsds.cbi.pku.edu.cn, accessed on 16 January 2022). The NCBI BATCH CD-search (https://www.ncbi.nlm.nih.gov/Structure/bwrpsb/bwrpsb.cgi, accessed on 26 January 2022) was used to examine the conserved domain structure based on the corresponding protein sequence, using the Pfam database. The conserved motifs were predicted by the MEME Suite tools (http://meme-suite.org, accessed on 6 February 2022, Bailey et al., 2009), the number of motif parameters was limited to less than 10 manually. The structural characteristics were evaluated with SWISS-MODEL online tools (Guex et al., 2009; Bertoni et al., 2017; Bienert et al., 2017; Waterhouse et al., 2018; Studer et al., 2020).

### 2.3 Chromosome distribution, gene duplication, syntenic relationship and expression analysis

The physical location information of *CsPRP* genes was obtained from the GCF_000004075.3_Cucumber_9930_V3_genomic database in NCBI, and all the identified genes were mapped to the cucumber chromosomes by TBtools V1.089 (Chen et al., 2020). Multiple Collinearity Scanning toolkit (MCScanX) with default parameters were used to analyze the gene duplication events (Wang et al., 2012). The expression of CsPRPs was analyzed using FPKM (fragments per kilobase of exon per million mapped fragments) values from RNA-seq datasets, including PRJNA307098, PRJNA321023, PRJNA80169, PRJEB7612, PRJNA214462, PRJNA263870, PRJNA271595, PRJNA285071, PRJNA292785, PRJNA312872, PRJNA319011, PRJNA376073, PRJNA258122, PRJNA345040, PRJNA380322, PRJNA382994, PRJNA388584, PRJNA419665, PRJNA431940, PRJNA437579, PRJNA438923, and PRJNA483118.

The cultivation of plant materials, sample collection, and processing of RNA-seq data were performed as described in the Cucurbit Expression Atlas (http://www.cucurbitgenomics.org/rnaseq/home).

### 2.4 Construction of expression vector, prokaryotic expression and silicon deposition characteristics of CsPRP1 and CsPRP3

To assess the binding activity of PRP, the coding sequence of PRP1 and PRP3 with or without the putative signal peptide was amplified from cucumber cDNA using primers containing *Nco*I and *Xho*I restriction site (as listed in **Supplementary Table 1**). The PCR products were then fused to a 6*His tag in the pET-28a expression vector and transformed into BL21(DE3) pLysS (CD701-02, Transgen, China). Expression of the fusion gene was induced with IPTG and the protein was purified by ProteinIso^®^ Ni-IDA Resin (DP111-01, Transgen, China).

The silicon deposition characteristics of CsPRP1 and CsPRP3 were then examined, with slight modifications to the method described by Kauss et al. (2003). This involved adding 10 μL of 0.5 mg/mL protein to 180 μL sodium phosphate/citrate buffer of different pH, followed by 10 μL 0.1M orthosilicic acid. After incubation at 25 °C for 30 min, the solution was centrifuged at 10000g for 10 min, the precipitation was collected and washed with distilled water. Silicon content in the precipitation was determined as previously described (Sun et al., 2020).

### 2.5 Subcellular localization of CsPRP1 and CsPRP3

To investigate the subcellular localization of CsPRP1 and CsPRP3, the coding sequences of them was amplified with primers containing *Sal*I and *BamH*I restriction sites. The PCR products were fused to the 5’ -end of enhanced green fluorescent protein (eGFP) under the CaMV 35S promoter in the pTF486 expression vector respectively (Primer sequences were listed in **Supplementary Table 1**). Expression of *CsPRP1* and *CsPRP3* in onion epidermal cells was performed by gene gun transduction as described previously (Duan et al., 2022). Onion epidermal cells transformed with empty vector (pTF486) was introduced as a positive control. The fluorescence signal was determined with a confocal laser scanning microscope (TCS SP8 SR Leica) at an excitation wavelength of 488 nm for GFP.

### 2.6 Construction of expression vector, *Arabidopsis thaliana* transformation and GUS staining

To investigate the expression character of *CsPRP1* and *CsPRP3*, the promoter (2000bp upstream of the start codon) of *CsPRP1* was isolated and inserted at the *Ec*oRI and *Nco*I sites, and that (2000bp upstream of the start codon) of *CsPRP3* was amplified and inserted at the *Kpn*I and *Nco*I sites of pCambia 1305, of which the promoters may direct the expression of *GUS* (Primer sequences were listed in **Supplementary Table 1**). The constructs were transformed into *Arabidopsis thaliana* (Columbia) following a floral dip protocol. To analyze *GUS* expression patterns, *Arabidopsis thaliana* seedlings and plants were left to soak in GUS staining buffer (composed of 2 mM X-Gluc in 0.5 M sodium phosphate buffer, pH 7.2, with 2 mM potassium ferrocyanide, 2 mM potassium ferricyanide, and 0.2% Triton X-100) overnight.

## 3 Results

### 3.1 Identification of *PRP* genes in cucumber genome

Iterative protein BLAST analysis was employed to identify the PRP genes in cucumber, using previously identified Arabidopsis PRP sequences as queries. The candidate *Cucumis sativus PRP* (*CsPRP*) genes were further scrutinized using BLASTp in the Swiss-Prot database and Batch-CDD tests. As a result, seven *CsPRP* genes were identified and their basic characteristics, including CDS lengths, amino acid sequence lengths, molecular weights, and isoelectric points, were analyzed and presented in **Table 1**. The localization of these proteins was predicted, with CsPRP2 being predicted to be located at the cell wall, CsPRP6 at the plasma membrane, CsPRP7 at the Cell membrane, Cytoplasm and Nucleus, and the other PRPs at the Nucleus. CsPRP6 and CsPRP7 were identified as hydrophilic proteins, while the other PRPs in cucumber were all hydrophobic. Additionally, a signal peptide with a length of 21–35 Aa was identified at the N terminal of the CsPRPs protein.

**Table 1:** Gene structure and protein characteristics of CsPRPs in cucumber.

Name	Gene ID	Gene symbol in NCBI	Chr	CDS length (bp)	Exon no.	Intron no.	Predicted location(s)	Number of amino acids	Molecular weight	Theoretical pI	Grand average of hydropathicity (GRAVY)	Cleavage site
CsPRP3	CsaV3_2G012830	LOC101203749	2	321	1	0	Nucleus.	106	12392.53	10.18	-0.889	pos.35 and 36
CsPRP1	CsaV3_2G012840	PRP1	2	279	1	0	Nucleus.	92	10905.78	10.12	-0.975	pos. 21 and 22
CsPRP2	CsaV3_2G012850	LOC101211209	2	1119	1	0	Cell wall.	372	40502.43	10.05	-0.592	pos. 25 and 26
CsPRP4	CsaV3_2G015730	LOC101204386	2	1650	2	1	Nucleus.	549	61416.17	9.52	-0.46	pos. 29 and 30
CsPRP5	CsaV3_7G000820	LOC101205516	7	1086	2	1	Nucleus.	361	40338.41	9.48	-0.618	pos. 21 and 22
CsPRP6	CsaV3_7G029410	LOC105436170	7	564	2	1	Cell membrane	187	20201.41	9.32	0.105	pos. 28 and 29
CsPRP7	CsaV3_7G029420	LOC105436171	7	519	2	1	Cell membrane. Cytoplasm. Nucleus.	172	18750.3	4.77	0.049	pos. 23 and 24

### 3.2 Phylogenetic analysis of the PRP families

**Figure 1:**
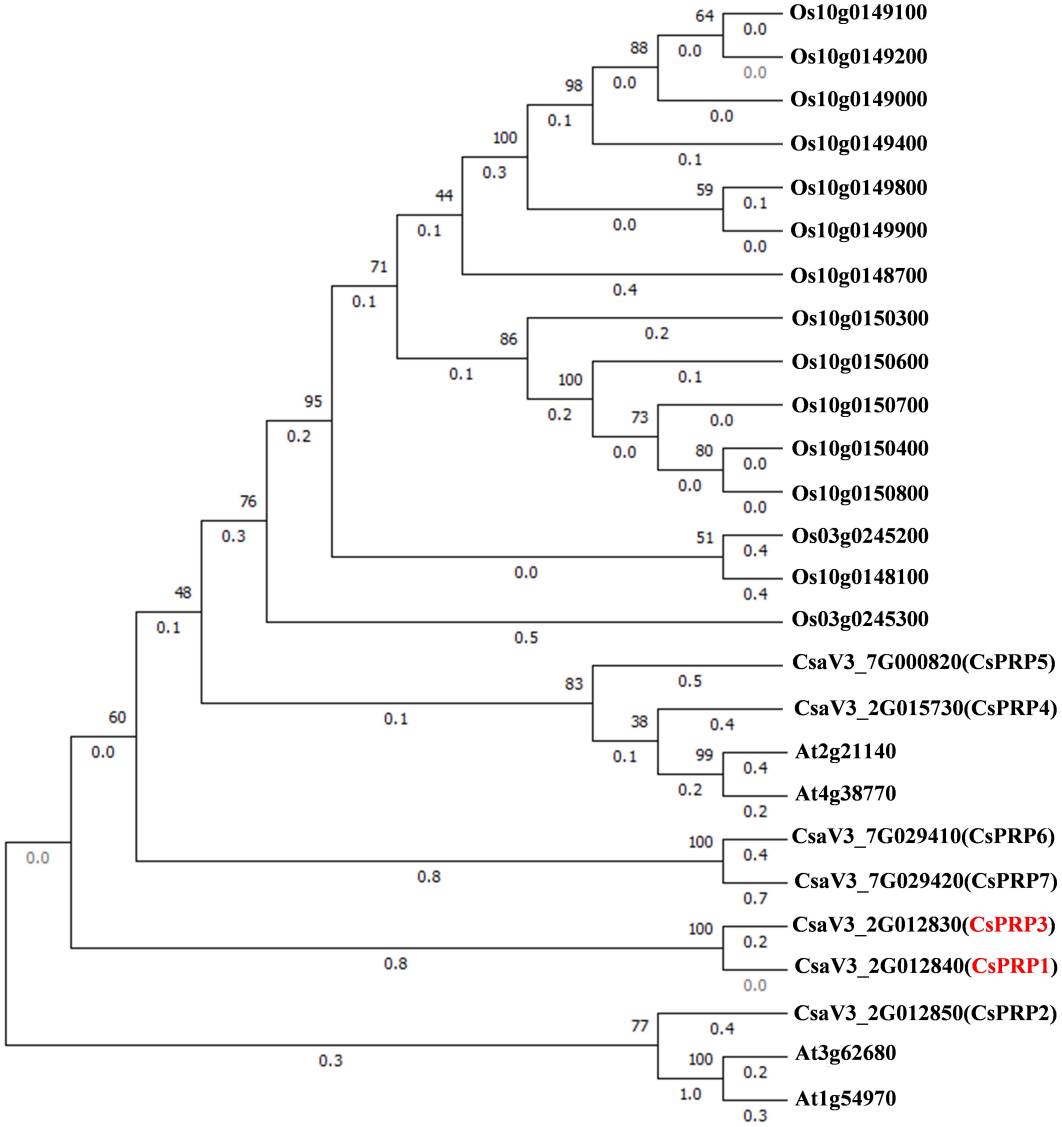
Phylogenetic analysis of PRP in cucumber, Arabidopsis and rice. Trees were built using amino acid sequences from cucumber (*Cucumis sativus*), *Arabidopsis thaliana* and rice (*Oryza sativa*). The phylogenetic tree was constructed using the maximum likelihood method with MEGA-X. Bootstrap was present on the left side of the node as a percentage. The branch length was marked below each branch.

An unrooted maximum likelihood tree was constructed using the MEGA software to analyze the phylogenetic relationship between PRPs from cucumber, Arabidopsis, and rice. These plants are representative of dicotyledonous and monocotyledonous model organisms. **Figure 1** illustrates that CsPRPs exhibit a closer relationship with AtPRPs than OsPRPs. Notably, CsaV3_2G012830(CsPRP3) is closely related to CsaV3_2G012840(CsPRP1).

### 3.3 Chromosome distribution, synteny analysis of *PRP* genes

**Figure 2:**
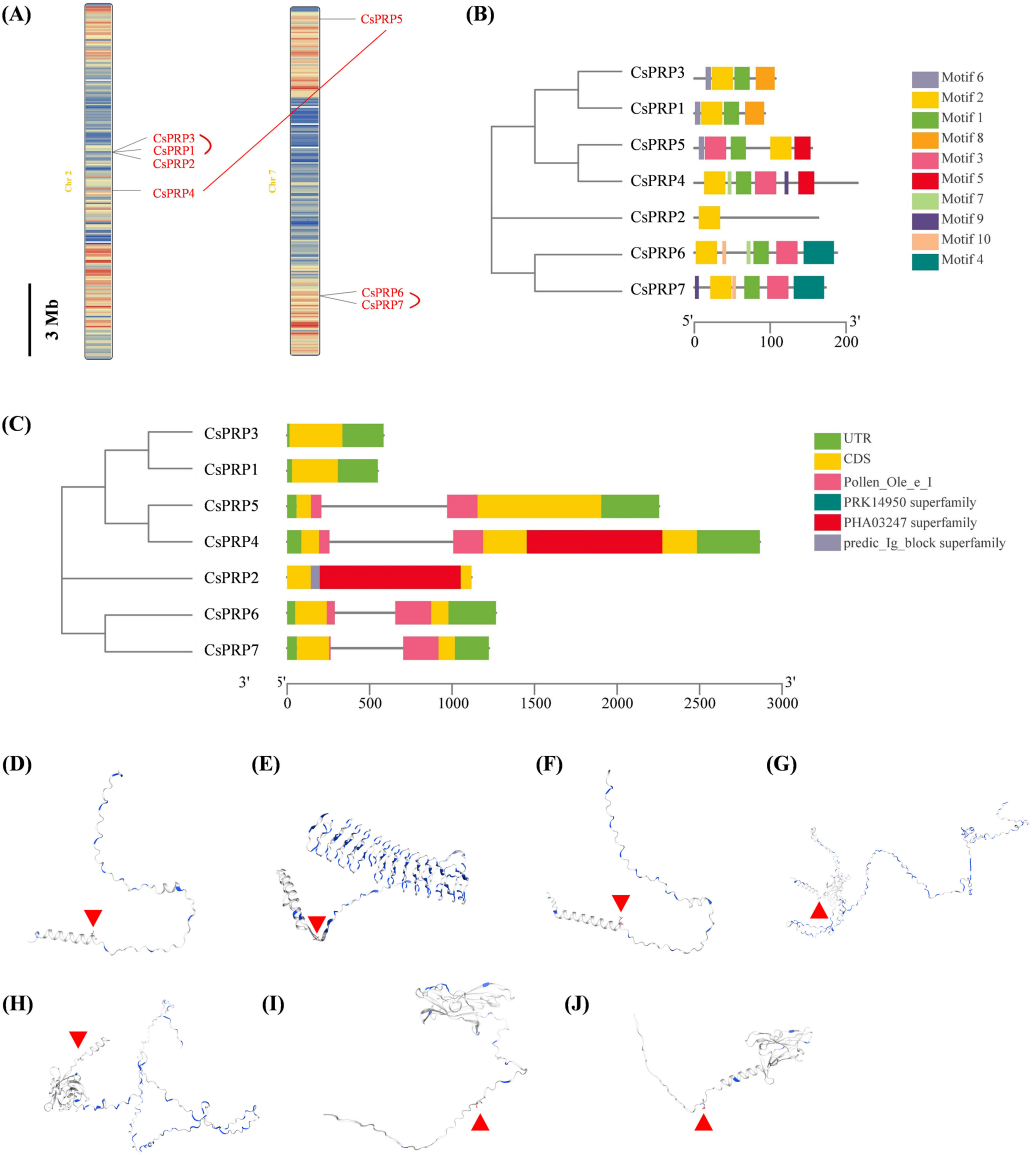
Chromosomal localization, gene replication events, gene structure, conserved domains and protein structure analysis of cucumber PRP family genes. Panel **(A)** shows the chromosomal localization and gene duplication events, panel **(B)** illustrates the gene structure, panel **(C)** highlights the conserved domains, and panels **(D-J)** display the protein structure analysis of CsPRP1 to CsPRP7, respectively. The red arrow indicates the predicted signal peptide cleavage site.

Chromosome distribution of *PRP* gene families was analyzed based on the physical location of the GCF_000004075.3_Cucumber_9930_V3_genomic database from the NCBI website. Gene duplication events of these genes including segmental and tandem duplications were analyzed using the MCScanX software. Results demonstrated that the *CsPRP* genes were distributed on two chromosomes-*PRP1*, *PRP2*, *PRP3* and *PRP4* on chromosome 2, and *PRP5*, *PRP6*, *PRP7* on chromosome 7. *CsPRP1*-*CsPRP3* and *CsPRP6*-*CsPRP7* were identified as tandem duplication, *CsPRP7* and *CsPRP5* were identified as segmental duplication (**Figure 2A**).

### 3.4 Gene, protein structure and conserved protein motif analysis of *PRP* gene families

The Gene Structure Display Server (GSDS) online program was used to analyze the intron-exon structure. Analysis on the conserved domain structure was based on the online program of NCBI BATCH CD-search tool (https://www.ncbi.nlm.nih.gov/Structure/bwrpsb/bwrpsb.cgi, accessed on 12 April 2020). The MEME search tool was employed to predict the conserved protein motifs. The results showed that the *CsPRP* genes had no more than one intron (**Figure 2B**). Pollen_Ole_e_I like (pfam01190) is the only member of the superfamily cl03128. DNA polymerase III subunits gamma and tau (PRK14950) is the only member of the superfamily cl36446. Large tegument protein UL36 (PHa03247) is the only member of the superfamily cl33720. Predic_Ig_block (TIGR04526) is the only member of the superfamily cl37462. CsPRP4, CsPRP5, CsPRP6 and CsPRP7 had Pollen_Ole_e_I domain, PHA03247 superfamily domain was conserved in CsPRP2 and CsPRP4, and predic_Ig_block superfamily domain only existed in CsPRP2 (**Figure 2C**). Alignment of CsPRPs demonstrated that the sequences differed and conserved motifs analysis indicated that CsPRP1 and CsPRP3 share similar motif pattern, both have four motifs. CsPRP4 and CsPRP5 share similar motif composition but the order was distinctive (**Figure 2**; **Supplementary Figures 1**, **2**), which may be associated with the segmental duplication event. The tertiary structure analysis indicates that CsPRP1 and CsPRP3 share a similar spatial conformation (**Figures 2D–J**), and it indicates that they may share similar biological functions.

### 3.5 Binding activity of CsPRP1 and CsPRP3 to silicon

**Figure 3:**
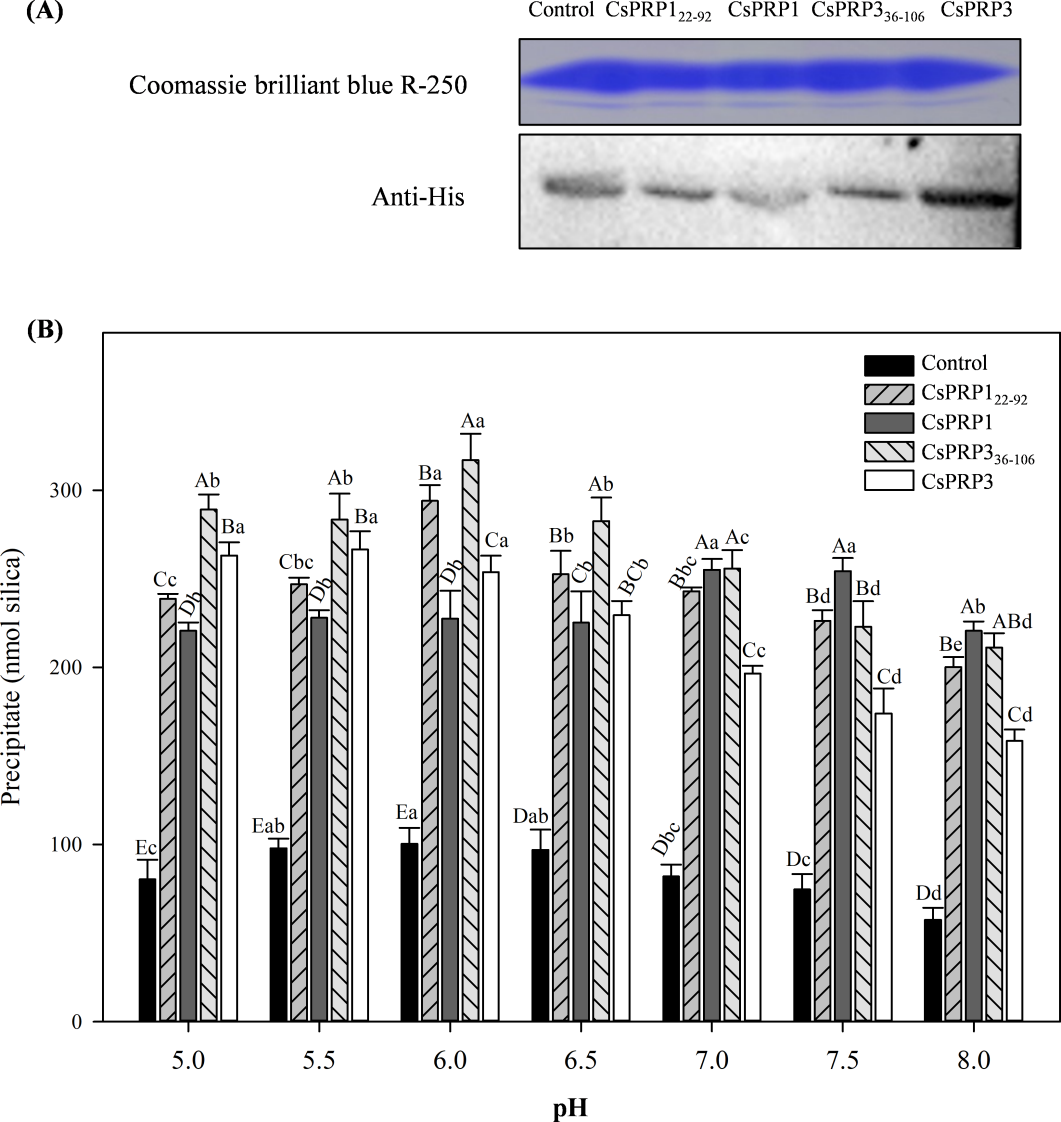
The binding characteristics of CsPRP1 and CsPRP3 to silicon. **(A)** displays the results of Coomassie blue staining and Western blot analysis using His-tag antibodies of purified proteins from various prokaryotic expression strains. **(B)** illustrates the binding capacity of the purified proteins from different prokaryotic expression strains to silicon under varying pH conditions. Under the same pH condition, different uppercase letters indicate significant differences at the *P*≦0.05 level, while different lowercase letters between the same carriers indicate significant differences at the *P*≦0.05 level.

This study achieved the prokaryotic expression of *CsPRP1* and *CsPRP3* in *Escherichia coli* BL21 by fusing their coding region sequences with a HIS tag protein on the pET28a vector, and then purified them with ProteinIso Ni IDA Resin. The binding experiment with silicon revealed that both CsPRP1 and CsPRP3, with or without a signal peptide, had a noticeable affinity for silicon, though their optimal pH values were different. CsPRP1 and CsPRP3 with signal peptide removed had the strongest binding capacity to silicon at pH 6.0, while CsPRP1 showed the most obvious binding at pH 7.0-7.5. The optimal pH of CsPRP3 binding to silicon was likely lower than 5.5 (**Figure 3**).

### 3.6 Intracellular localization of CsPRP1 and CsPRP3

**Figure 4:**
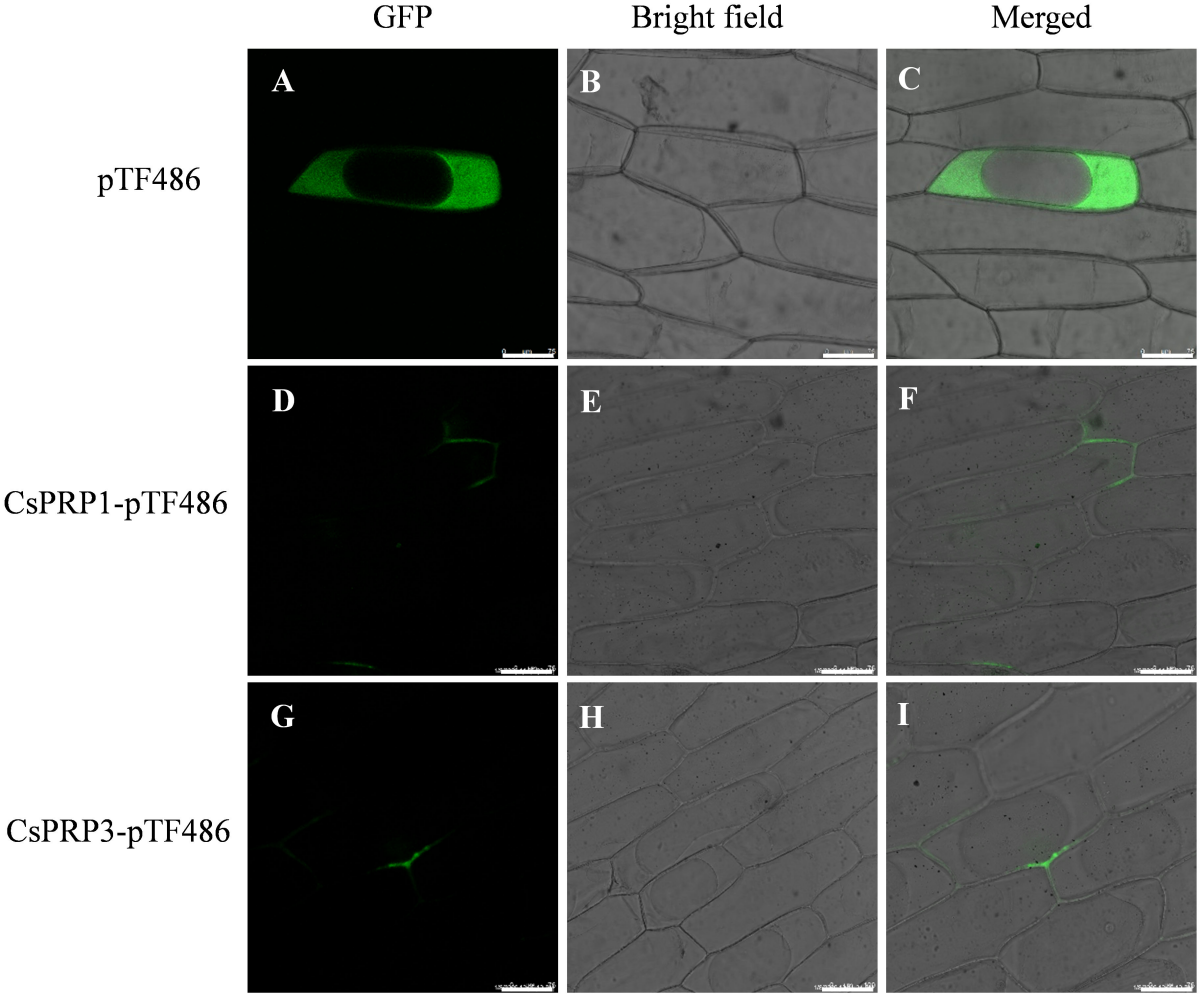
The subcellular distribution of CsPRP1 and CsPRP3. The fluorescent signal distribution following the transformation of onion epidermis with empty vectors is illustrated in **(A–C)**. The subcellular localization of CsPRP1 and CsPRP3 in onion epidermis is shown in **(D–F, G–I)**, respectively. **(A, D, G)** display images taken in the green fluorescent channel, while **(B, E, H)** show bright-field images. **(C, F, I)** present overlays of images from the green fluorescent channel and bright-field. Bar=75 μm.

In this research, the coding sequences of CsPRP1 and CsPRP3 were fused with green fluorescent protein (GFP) and introduced into onion epidermal cells using the gene gun method. The results showed that the empty vector control caused the interior of the onion cells to emit green fluorescence, except for the large vacuoles. However, the proteins of CsPRP1 and CsPRP3 were specifically localized on the cell walls and exhibited distinct polarity distribution patterns. The presence of green fluorescence on adjacent cell walls suggested that these proteins may have the ability to move between neighboring cells (**Figure 4**).

### 3.7 Expression characterization of *PRP* in cucumber

**Figure 5:**
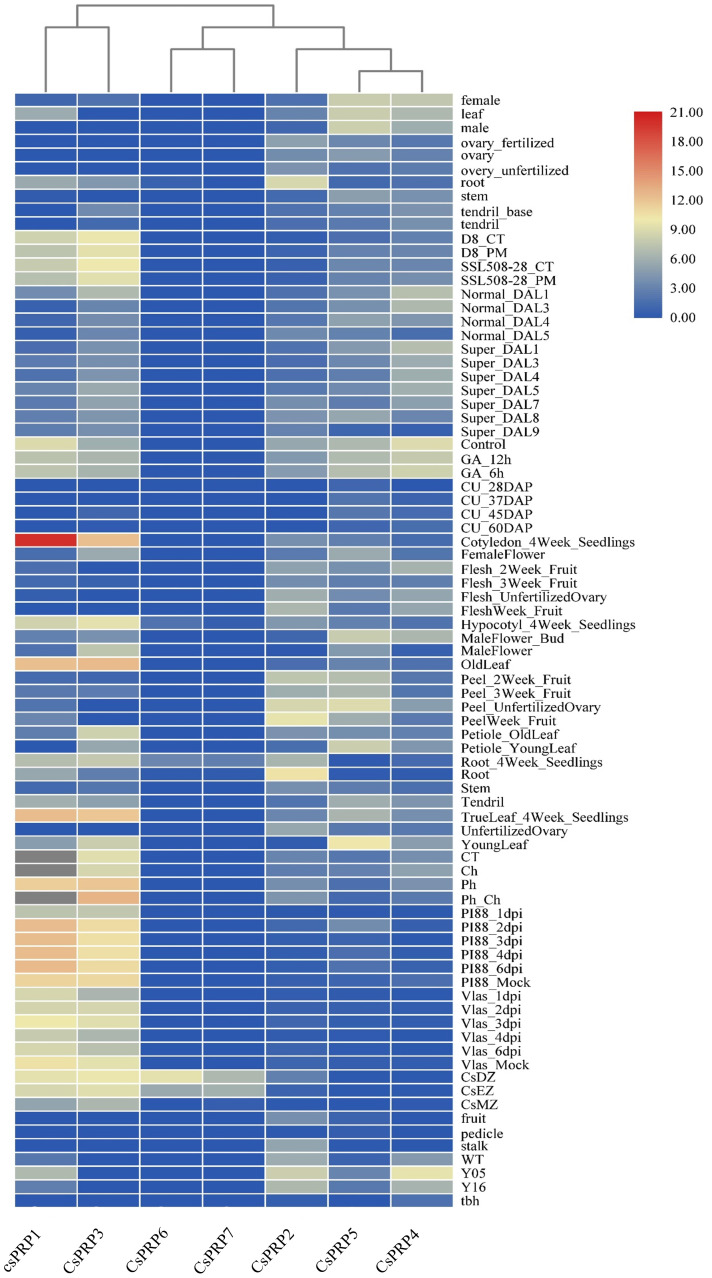
Expression pattern of cucumber PRP family. The sampling locations used for expression analysis in the figure include roots, leaves, seeds, ovaries, flowers, fruits, pedicels, and stems. The data presented is derived from RPKM that has been exponentially transformed and is represented by colors ranging from red to blue, with red indicating the highest expression and blue indicating the lowest. The abbreviations used in the figure are CT (control), DAP (day after pollination), DAL (ovary 4–5 days after flowering), Tbh (tiny branched hair mutant), Y05 (fruit thorns on 0.5 cm large fruits), Y16 (fruit thorns on 1.6 cm large fruits), WT (wild type cucumber), CsDZ (Root differentiation zone), CsEZ (Root elongation zone), CsMZ (Root meristematic zone), Ch (treated with chrysophanol), Ph (treated with formaldehyde), GA_ 12 hours (treated with gibberellin for 12 hours), and GA_ 6 hours (treated with gibberellin for 6 hours).

Expression investigation of PRP family genes from high-throughput sequencing data showed that *CsPRP6* and *CsPRP7* had similar expression patterns, with expression only detected in the root differentiation and elongation regions, indicating their potential role in cucumber root development. *CsPRP1* and *CsPRP3* were found to be expressed in cucumber roots, leaves, and flower organs, but not in stems and fruits. The expression of *CsPRP2*, *CsPRP5*, and *CsPRP4* varied across different tissue parts of cucumber, with *CsPRP2* mainly expressed in roots and fruits, and *CsPRP5* and *CsPRP4* mainly expressed in leaves, fruits, and flower organs. Additionally, under GA treatment conditions, the expression of *CsPRP1* and *CsPRP4* was significantly reduced, suggesting their involvement in gibberellin-mediated signal transduction and growth and development in cucumber (**Figure 5**).

**Figure 6:**
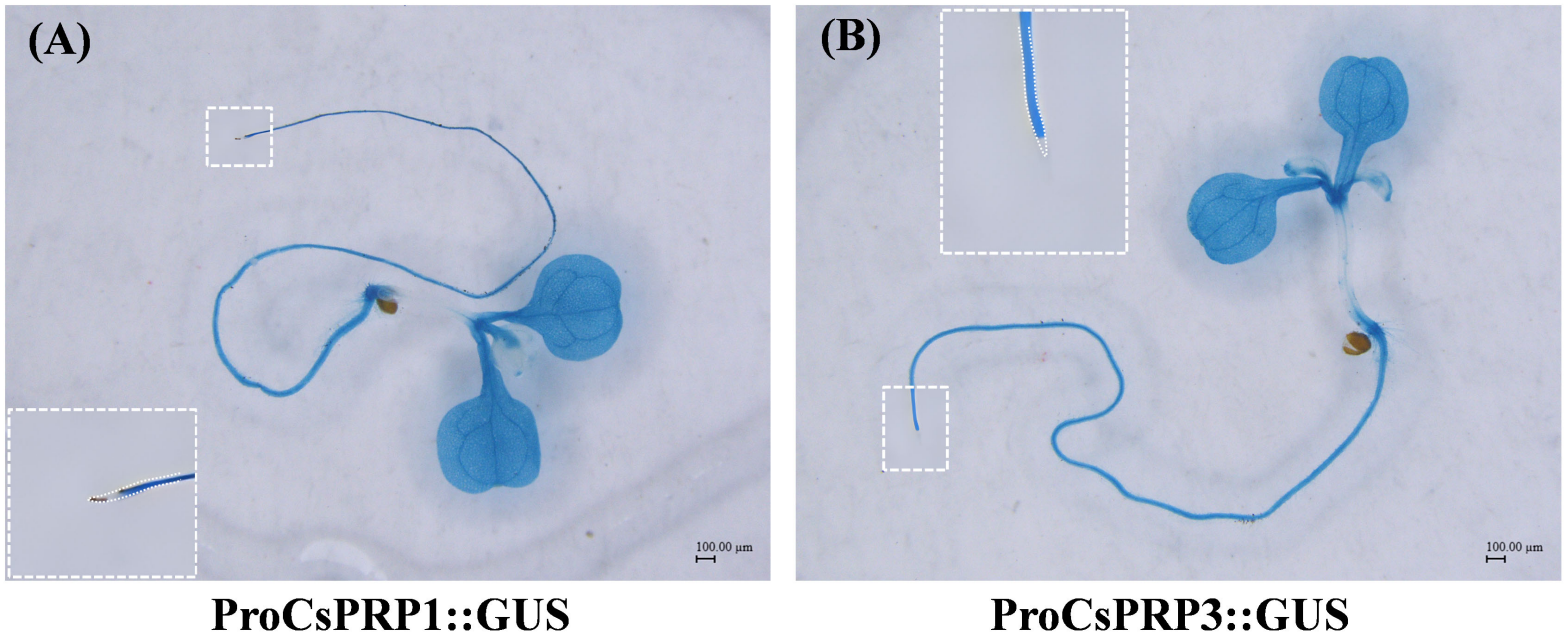
GUS staining of ProCsPRP1::GUS and ProCsPRP3::GUS transgenic Arabidopsis at seedling stage. The staining of ProCsPRP1::GUS transgenic Arabidopsis seedlings was shown in **(A)** and that of ProCsPRP3::GUS transgenic seedlings was demonstrated in **(B)**.

The promoter regions of *CsPRP1* and *CsPRP3* were isolated and ProCsPRP1::GUS and ProCsPRP3::GUS expression vectors were constructed and used to generate transgenic Arabidopsis. GUS staining at different growth stages of the transgenic Arabidopsis revealed that blue was observed in the mature leaves and roots of ProCsPRP1::GUS transgenic Arabidopsis seedlings, while no GUS gene expression was detected in the young leaves, stems, and root tips (**Figure 6A**). The same pattern was observed in ProCsPRP3::GUS transgenic Arabidopsis seedlings (**Figure 6B**). These findings suggest that *CsPRP1* and *CsPRP3* are likely only expressed in mature leaves and roots during the seedling stage.

**Figure 7:**
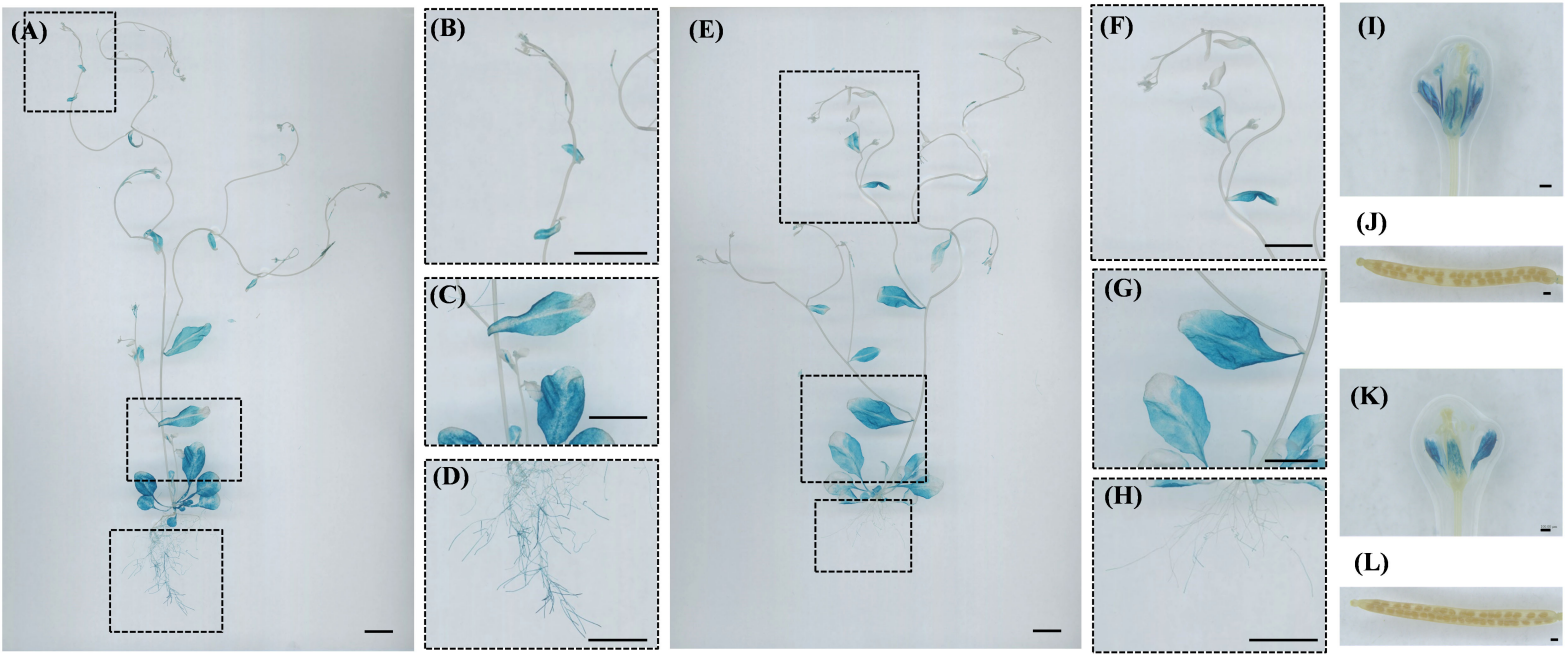
GUS staining of ProCsPRP1::GUS and ProCsPRP3::GUS transgenic Arabidopsis at reproductive stage. The staining results of reproductive growth stage Arabidopsis plants with ProCsPRP1::GUS transgene are shown in images **(A–D, I–J)**, while images **(E–H, K, L)** depict the staining results of different plant parts with ProCsPRP3::GUS transgene. **(A, E)** show staining of the entire plant, I and K show staining of flowers in ProCsPRP1::GUS and ProCsPRP3::GUS transgenic Arabidopsis, **(J, L)** show staining of siliques, and **(B–D, F–H)** were the amplification of the insets in **(A-E)**. Bar=1 cm in **(A–H)**, and 100 µm in **(I-L)**.

During the reproductive growth stage, *CsPRP1* is primarily expressed in the leaves, roots, petals, and stamens of the plant. The expression of *CsPRP1* in the leaves increases with age, although it is scarcely detected in young leaves. In the roots, expression is limited to the lowest-level lateral roots (**Figures 7A–D**). *CsPRP1* is also expressed in the petals and stamens (**Figure 7I**). *CsPRP3* exhibits a similar expression pattern to *CsPRP1* in the leaves. In the roots, it is only expressed in the lowest lateral roots, but at significantly lower levels than *CsPRP1* (**Figures 7E–H**). *CsPRP3* is expressed solely in the petals of the flowers, and neither gene is expressed in the pods or seeds (**Figures 7J–L**).

## 4 Discussion

Plant uptake silicon from earth liquid by root, and transport it upward via apoplastic and/or symplastic pathways (Coskun et al., 2019), which leads to an accumulation of silicon that is distinct in terms of content in different plants and increases resistance to stresses (Mitani-Ueno et al., 2016). Previous research has suggested that an organic environment, containing carbohydrates, callose, proteins, lipids, phenolic compounds, and metal ions, may be involved in biosilica formation (Harrison, 1996; Currie and Perry, 2007; Law and Exley, 2011; Sahebi et al., 2015). Cucumber is a typical high silicon accumulator in dicotyledons and the proline - rich protein family was analyzed bioinformatically, their expression and silicon binding characteristics were also investigated.

Proline - rich Protein (PRP) was initially discovered in the breakdown products of cell walls (Lamport and Clark, 1969). Early research suggested its role in the formation of cell walls, highlighting its significance as a key component in the primary and secondary walls of plant tissues, as well as in the symbiotic root nodule cell walls of leguminous plants (Lamport and Clark, 1969). Kauss et al. (2003) further identified the potential role of PRP in silicon deposition in cucumber. In this study, seven *PRP* genes were identified from the cucumber genome, including two sets of tandemly repeated genes: *CsPRP1* - *CsPRP3* and *CsPRP6* - *CsPRP7*. These two pairs of tandem repeat genes exhibit high similarity in terms of internal gene structure, composition of conserved domains and gene expression characteristics, suggesting that they may possess similar or identical functions in cucumber.

Silicon in the external environment of plants has been found to form associations with organic compounds (Samuels et al., 1991; Bathoova et al., 2018; Carneiro-Carvalho et al., 2020; Zexer et al., 2023). Cell wall constituents including polysaccharides, pectin, and lignin harbor abundant hydrogen bond-forming sites, prompting the notion that all major cell wall polymers are implicated in regulating silica deposition (Lux et al., 2020). Emerging evidence also highlights the functional roles of cell wall proteins in this process. A case in point is the identification of Siliplant1 in sorghum, which has been shown to mediate silica deposition specifically within leaf silica cells (Kumar et al., 2020). Kauss et al. (2003) demonstrated that the capacity of CsPRP1 to bind to silicic acid was not dependent on its protein sequence, but associated with the clustering of positively charged amino acids on its peptide, which was determined by comparing the binding abilities of different peptide segments of CsPRP1 to silicic acid. The binding capacity of CsPRP1 and CsPRP3 to silicon, as measured in this study, was found to be approximately two to three times that of some peptides in Kauss et al. (2003) study. This could be due to the cumulative effect of the positive amino acid clusters in the entire length of CsPRP1 and CsPRP3. Additionally, the protein extracted from the control sample in this study also exhibited some binding properties to silica, which may be attributed to the presence of Histidine.

Generally, the pH of extracellular liquid in most tissues and organs is acidic (Felle, 2001; Raghavendra et al., 2023). Our studies demonstrate that both signal peptide-containing and signal peptide-removed forms of CsPRP1 and CsPRP3 exhibit silicon-binding activity across the pH range of 5.0–8.0, with the signal peptide-removed variants showing highest affinity for silicon at pH 6.0. Moreover, within the pH range of 5.0–6.5, the silicon-binding capacity of the signal peptide-removed CsPRP1 and CsPRP3 is lower than that of their signal peptide-retaining counterparts. These findings indicate that both forms of CsPRP1 and CsPRP3 possess silicon-depositing ability, but they likely differ in the sites and conditions under which they exert maximal silicon-depositing activity. The mature, signal peptide-removed forms of CsPRP1 and CsPRP3 are likely to be primarily involved in silicon deposition between plant cell walls. Notably, the cytoplasmic pH of plant cells (7.0–7.5) coincides with the optimal pH for silicon-depositing activity of signal peptide-retaining CsPRP1, suggesting that this form may be involved in silicon sequestration within the plant cytoplasm (Falkner and Horner, 1976; Li et al., 2024). Silicon is mainly found in the extracellular space of plants cell, such as the epidermal cells of leaves in Poaceae plants (Jones and Handreck, 1967). Kumar et al. (2020; 2021) investigated the cellular processes of protein-dependent mineralization in plants, diatoms and sponges, and concluded that in grass silica cells, silica deposition occurs outside the cell membrane when the cells secrete the mineralizing protein into the apoplasm, which is abundant in silicic acid (the mineral precursor molecules). This study found that CsPRP1 and CsPRP3 were located on the cell wall at the subcellular level (**Figure 4**), and the proteins encoded by the two genes had a strong affinity for silicon. This suggests that CsPRP1 and CsPRP3 may be involved in the deposition of silicon in the cell walls of cucumber plants. Additionally, the polarity distribution patterns also suggest that the two proteins may have specialized roles in the process of silicon deposition.

In this study, ProCsPRP1::GUS and ProCsPRP3::GUS expression vectors were created and used to genetically transform Arabidopsis. GUS staining of the transgenic plants revealed that *CsPRP1* and *CsPRP3* had similar expression patterns, with the highest expression in mature leaves and roots at the seedling stage, and in mature leaves, roots and petals after flowering. Additionally, the expression level in leaves increased with age, and in roots, they were only expressed in the lowest lateral roots (**Figure 7**). The expression patterns of *CsPRP1* and *CsPRP3* were similar to silicon deposition observed in cucumber. For instance, Adatia and Besford (1986) studied the distribution of silicon in cucumber leaves and found that the silicon content decreased from old leaves to young leaves, which is also observed by Sangster (1970) and this suggests that the expression of *CsPRP1* and *CsPRP3* in different parts of cucumber may be related to the accumulation of silicon in that area.

Previous studies have indicated that the deposition of silica in plants is active and physiologically regulated (Kumar et al., 2017); however, the underlying molecular mechanisms remain largely elusive. Research on diatom has suggested that Silicanin-1 and Silicalemma Associated Proteins (SAPs) play a role in silica architecture (Tesson et al., 2017; Görlich et al., 2019), and homologous genes in higher plants may have potential for silicon deposition research. Recently, Zexer et al. (2023) suggested that carbonyl-rich lignin monomers in the apoplast may be involved in plant silicon deposition, lignin extension bonds bind silanols, nucleating silica aggregates near extrusion loci. Although some genes in rice and wheat have been speculated to be associated with silicon deposition, the exact functions of these genes remain to be elucidated (Peleg et al., 2010; Yu et al., 2020). This study reveals that *CsPRP1* and *CsPRP3* are predominantly expressed in the mature leaves and roots during the seedling stage, and in the leaves, roots, petals, and stamens at maturity. GFP fusion assays in onion epidermal cells demonstrated their specific localization to the cell wall. Silicon binding assays further showed that both proteins bind to silicon, with distinct optimal pH values. These findings provide new insights into the molecular mechanisms of silicon deposition in plants and may aid in enhancing crop stress resistance.

## Data Availability

The original contributions presented in the study are included in the article/**Supplementary Material**. Further inquiries can be directed to the corresponding author/s.
