# Correction: Characterization of two proline-rich proteins involved in silicon deposition in *Cucummis sativus*

**DOI:** 10.3389/fpls.2025.1695193

**Published:** 2025-09-09

**Authors:** Hao Sun, Feijuan Gao, Xiaoping Kong, Zhen Jiao, Zhongfang Tan, Jie Wu, Bochao He, Yaoke Duan

**Affiliations:** ^1^ National Key Laboratory of Cotton Bio-breeding and Integrated Utilization, School of Agricultural Sciences, Zhengzhou University, Zhengzhou, China; ^2^ Henan Key Laboratory of Ion-Beam Green Agriculture Bioengineering, School of Agricultural Sciences, Zhengzhou University, Zhengzhou, China; ^3^ Shaanxi Engineering Research Center for Vegetables/College of Horticulture, Northwest A&F University, Yangling, China; ^4^ Xining Vegetable Technical Service Center, Xining, China; ^5^ College of Agriculture and Animal Husbandry, Qinghai University, Xining, China

**Keywords:** silicon deposition, proline-rich protein, cucumber, biogenic silica, silicification

Author “Hao Sun” was erroneously assigned as a corresponding author. The only corresponding author is “Yaoke Duan”.

The original version of this article has been updated.

