# Supplementary material for: Characterization of two proline-rich proteins involved in silicon deposition in Cucummis sativus
Source: Front Plant Sci. 2025 Aug 28;16:1664009. doi: 10.3389/fpls.2025.1664009 (PMC12456188; doi:10.3389/fpls.2025.1664009)
Supplement: Supplementary file 1 [file DataSheet1.docx]

Supplementary Material

# Supplementary Figures and Tables

##
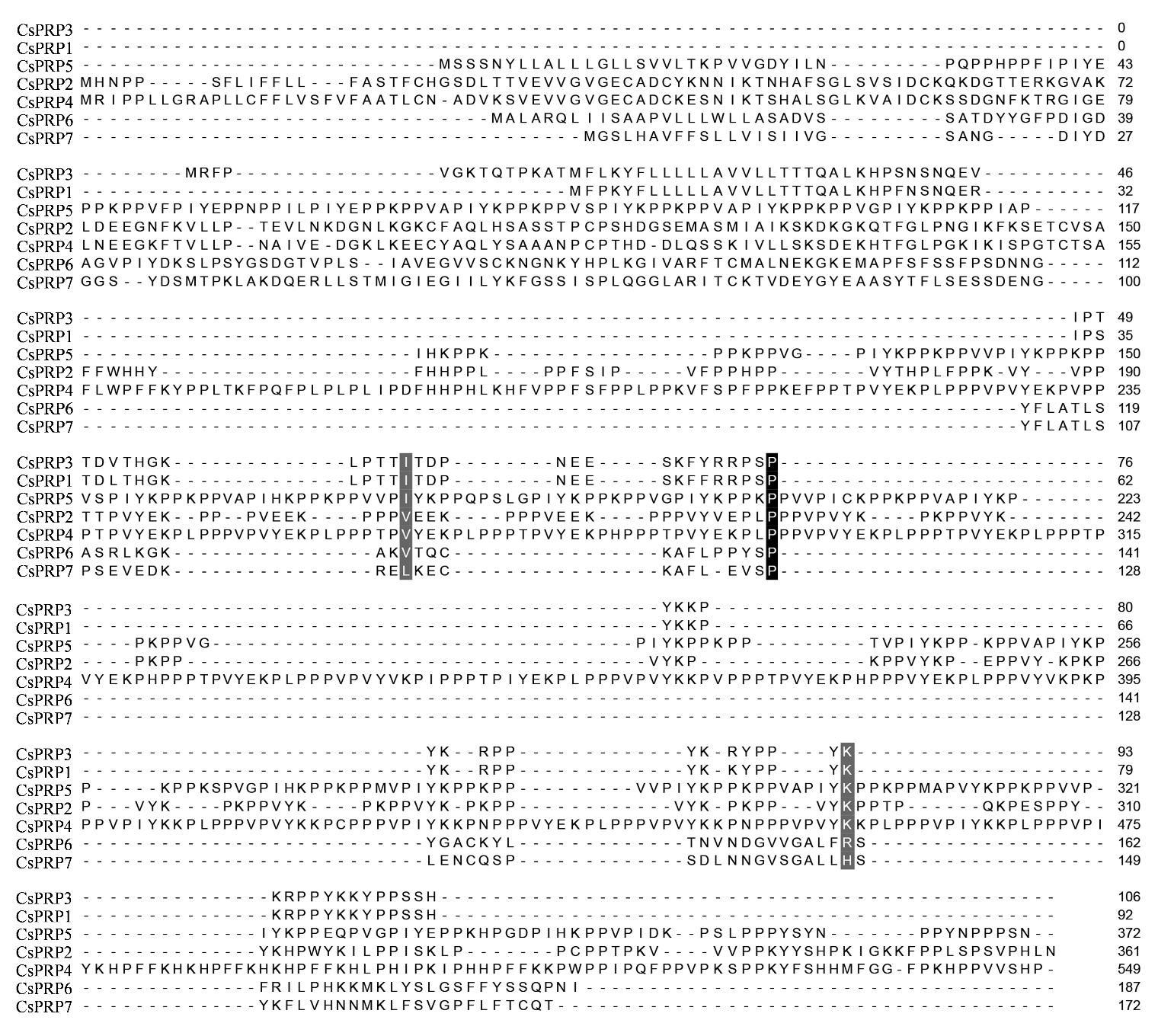
Supplementary Figures

**Supplementary Figure 1.** Alignment of the cucumber PRP family.


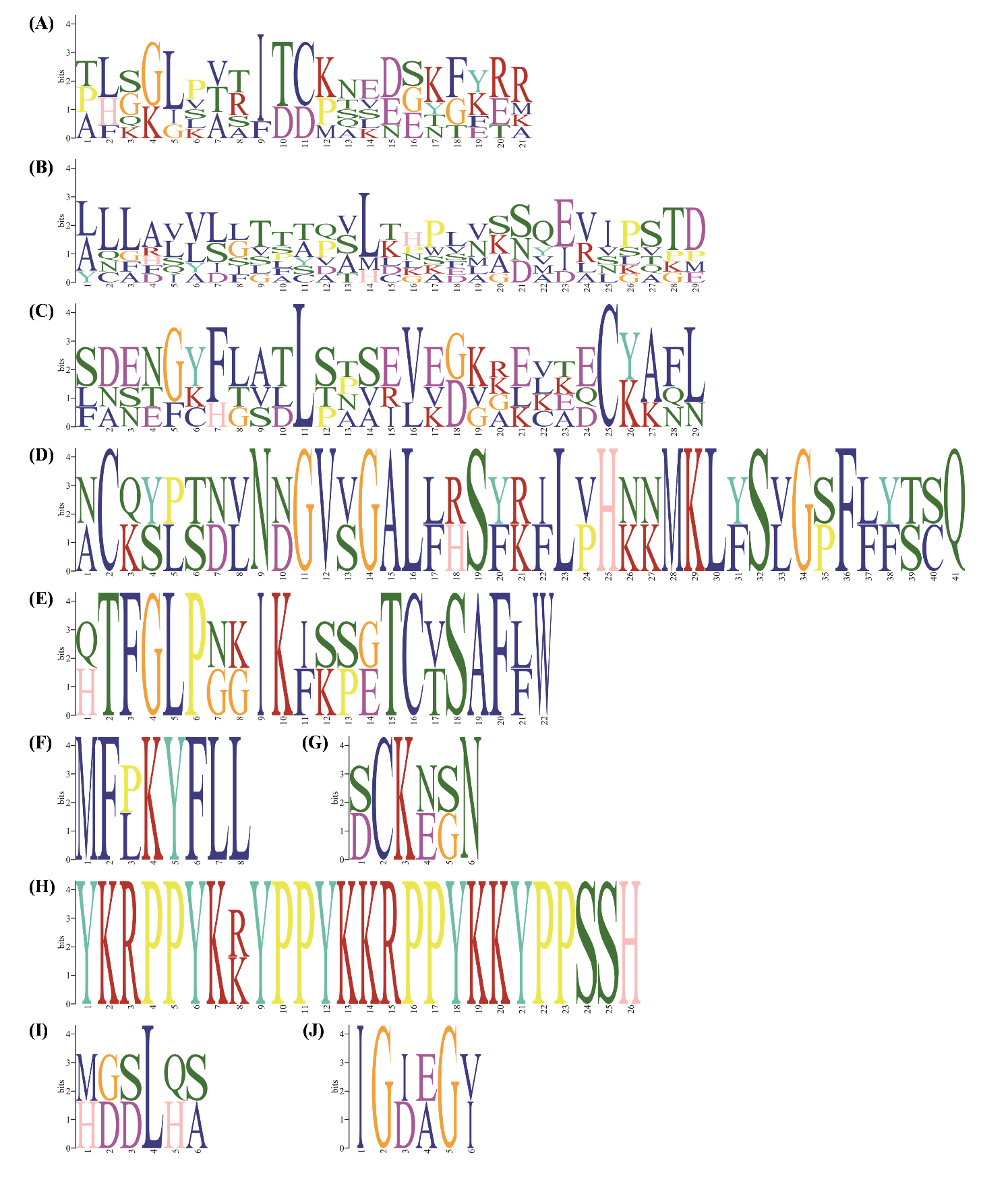


**Supplementary Figure 2.** Sequences of motifs identified in cucumber PRP family.

## Supplementary Tables

**Supplementary Table 1** Primers introduced in this study

| Forward primer | Reverse primer | Description |
| --- | --- | --- |
| CTTTAAGAAGGAGATATACCATGGGGCTGAAGCATCCCTTCAACTCCAAC | GTGGTGGTGGTGGTGGTGCTCGAGGTGAGAAGAGGGTGGGTACTTCTT | For the constrction of PRP1_22-92_ in pET28a |
| CTTTAAGAAGGAGATATACCATGGGGATGTTTCCCAAATACTTTCTTCTC | as above | For the constrction of PRP1 in pET28a |
| CTTTAAGAAGGAGATATACCATGGGGCTGAAACACCCCTCCAACTCCAAC | GTGGTGGTGGTGGTGGTGCTCGAGGTGGGAAGATGGTGGGTACTTC | For the constrction of PRP3_36-106_ in pET28a |
| CTTTAAGAAGGAGATATACCATGGGGATGAGGTTCCCAGTAGGAAAAAC | as above | For the constrction of CsPRP3 in pET28a |
| ACGCgtcgacATGTTTCCCAAATACTTTCTTCTCC | CGggatccGTGAGAAGAGGGTGGGTACT | For the constrction of CsPRP1 in pTF486 |
| ACGCgtcgacATGAGGTTCCCAGTAGGAAAAA | CGggatccGTGGGAAGATGGTGGGTACTT | For the constrction of CsPRP3 in pTF486 |
| gGAATTCTAATTTAAATATACAAATTATTAAGGGTTGACA | CATGccatggTGTGGCTTTTCGAGTTTGAG | For the constrction of CsPRP1 promoter in pCambia1305 |
| GGggatccAGATTTTTGTGATGTCTAGGAGCAA | CATGccatggACGTTGTGGCCTTTTATAGCATTTT | For the constrction of CsPRP3 promoter in pCambia1305 |
